# The Association between Self-Rated Health and Health Self-Management Ability of Healthcare Undergraduates: The Chain Mediating Roles of eHealth Literacy and Resistance to Peer Influence

**DOI:** 10.3390/ijerph192114501

**Published:** 2022-11-04

**Authors:** Guangyi Xu, Yanhong Xu, Xiaomin Tu, Shuaijia Hao, Ting Liu

**Affiliations:** School of Nursing, Qingdao University, Qingdao 266071, China

**Keywords:** health self-management ability, eHealth literacy, peer influence, self-rated health, healthcare undergraduates, China, mediation effect

## Abstract

Health self-management is important for healthcare undergraduates who are at the late adolescent or early adulthood stage, and will play an important part in health promotion for the general population. Previous research has shown that perceived health status affects health self-management. However, few studies have uncovered the mechanism between self-rated health and health self-management among healthcare undergraduates. Based on social ecology theory and Pender’s health promotion model, this study aimed to explore the associations between health self-management ability, self-rated health, eHealth literacy and resistance to peer influence of healthcare undergraduates, with a focus on identifying the mediating effects of eHealth literacy and resistance to peer influence. A cross-sectional study was conducted among 515 healthcare undergraduates in Eastern China between July and September 2021. Serial mediation analysis was performed using Haye’s Model 6 PROCESS macro to examine the mediating effects. The study found that health self-management ability was significantly and positively correlated with eHealth literacy, resistance to peer influence and self-rated health. Self-rated health had a direct and positive predictive effect on health self-management, with a direct effect value of 0.654. eHealth literacy and resistance to peer influence played both an independent mediating and a chain-mediating role in the mechanism of self-rated health affecting health self-management among healthcare undergraduates, with indirect effect values of 0.085, 0.101, and 0.013, respectively. The results suggest that eHealth literacy and resistance to peer influence could be intervention targets in programs for improving these students’ health self-management ability.

## 1. Introduction

Undergraduate students are at the stage of late adolescence or early adulthood, during which their establishment of healthy habits and health-related behaviors shape their current and future health [1,2]. Meanwhile, this population will probably transit from pediatric to adult-centered health care settings that involve gradual acquisition of health self-management skills [3]. For healthcare undergraduates, most will become health professionals who commit to promoting people’s health in the future. Research shows that medical students in China, U.S.A. and Australia with healthy behaviors were more likely to hold favorable attitudes towards and confidence in practicing preventive counseling than those with unhealthy behaviors [4]. Therefore, preparing healthcare students to develop health self-management abilities and engage in healthy behaviors will contribute to enhancing both their own health and positive health outcomes for the general population.

Studies have reported that health self-management was suboptimal among college healthcare students, which can manifest as various physical, mental and social health problems such as sedentary behaviors, binge drinking, eating disorders [5,6], stress, depression, and loneliness [2,7]. For instance, many nursing and medicine students did not achieve recommended levels of physical activity in the U.K., which increases their health risk and may influence their health promotion practice with clients [5]. Notably, a study found that health science students had poorer dietary behaviors than non-health science students in 17 low and middle income countries [6]. Medical students reported higher levels of psychological distress than the same-age peers [8]. These studies suggest that health self-management is paramount for healthcare students, which contributes to their own health as well as improved quality of care in future healthcare practice.

## 2. Theoretical Background and Hypotheses

Pender’s health promotion model and Bronfenbrenner’s social ecology theory were used as the theoretical background of this study.

Pender’s health promotion model (HPM) posits that situational influences are predictors of an individual’s health-promoting behaviors [9]. Situational influences refer to individuals’ perceptions and cognition of any situation or context that can facilitate or prevent the person’s behavior. Perceived health status is a type of situational influence, and closely linked with health behaviors. For instance, Korean older adults perceiving good health status reported a higher level of health-promoting behaviors than those who perceived poor health status [10]. Health self-management strategy was independently associated with five health statuses (i.e., physical, mental, social, spiritual and general health status) for the general South Korean population [11]. These findings indicate that perceived health status may be a factor contributing to health self-management for healthcare undergraduates. 

The social ecology theory states that individuals’ health is affected by multiple and interacting factors situated in multi-layered systems [12]. For instance, factors within the macro-system (e.g., the cultural, societal, and economic fields) exert their influence through micro-level systems (e.g., peers, family, school, and neighborhood fields) to affect behavior [13]. For undergraduates as adolescents or emerging adults, peer is one of the compelling sources of social influence on health behaviors. Pender also proposed that interpersonal influences (e.g., family, peers, and providers) affect decision-making about health-promoting behaviors [9]. Previous research revealed that descriptive peer norms demonstrated stronger associations with both adolescent health-promoting behaviors (e.g., physical activity) and health-impairing behaviors (e.g., sedentary habits) than parental descriptive norms [14]. Roberson et al. [15] reported direct and indirect effects of peers on drinking behavior among college students. A qualitative study revealed that peer interaction was a source of stressor facing medical students [8]. Resistance to peer influence could function as a buffer between peer influence and risky decision-making [16]. Taken together, peers may play a considerable role in the health self-management of healthcare undergraduates. However, few studies have explored the relationship between peer influence and health self-management for healthcare undergraduates.

The social ecology theory also suggests that digital technologies located in the exosystem is a proximal factor influencing health behaviors of young people [12]. Digital technologies such as smartphone applications and mass media constitute a context for the daily interaction of adolescents that can promote both health-compromising and health-enhancing behaviors [17]. In HPM, Pender emphasizes that the use of technological advances is effective to achieve healthy life behaviors and positive health outcomes [18]. With the rapid development of information and communication technology in recent years, globally there has been a rise of health self-management through the acquisition and use of health information from various digital sources [19,20]. College students are more likely to often use the internet for health information over other sources [21]. In a qualitative study, Chinese college students perceived eHealth tools as convenient in health self-management and usually used them to monitor their own health. In particular, medical major college students actively accessed online health information as references to solve their health problems [22]. However, there is a notable risk of exposure to unreliable health information, a difficulty in identifying the correct information available online, or misuse of health information, which result in adverse effects such as anxiety [23]. Therefore, eHealth literacy is required for the ability to search, understand, and evaluate reliable health information available via electronic resources [24].

eHealth literacy was found to be a major determinant of health self-management among patients with chronic diseases [25,26]. For patients with chronic kidney disease, eHealth literacy exerted impact on all aspects of their self-management: self-integration, problem solving, and seeking social support [25,26]. Patients suffering from chronic obstructive pulmonary disease with sufficient eHealth literacy reported positive experiences of eHealth resources in improving their disease self-management skills (e.g., exercises and breathing techniques) and boosting their confidence in their ability to manage their disease [20]. Other studies demonstrate that eHealth literacy was significantly and positively associated with health lifestyle behaviors (e.g., health responsibility, stress management) among undergraduate nursing students in Turkey [27] and in Korea [19]. Accordingly, eHealth literacy may play a protective role in promoting health self-management among healthcare undergraduates.

For current healthcare undergraduates, digital technologies and peers are two significant sources of influence on health self-management. The social ecology theory highlights the interaction of factors influencing individuals’ health [12]. Hence, eHealth literacy and peer influence may interrelate to jointly affect healthcare undergraduates’ health self-management. Simultaneously, research has established relationships among self-rated health, eHealth literacy and peers in various populations. For instance, a large-scale national study concluded that self-rated health was associated with health literacy among Chinese residents aged 15–69 years [28]. Resistance to peer influence was positively correlated with self-rated health among Chinese healthcare undergraduates [2]. A qualitative study revealed that health status was a motivator or excuse for not visiting the chronic obstructive pulmonary disease web [20]. Specifically, some participants claimed that feeling too well would not motivate them to search for information while others perceived that they had to feel good to be able to take in advice and information from electronic resources [20]. This contradictory experience indicates that the association between health status and eHealth literacy needs to be further examined. 

Based on social ecological theory, Pender’s HPM, and extant literature, we hypothesized that self-rated health would be correlated with health self-management among undergraduate healthcare students. Further, we hypothesized that eHealth literacy and resistance to peer influence would mediate the relationship between self-rated health and health self-management. The present study will expand prior research on the relationship between self-rated health and health self-management by examining potential mechanisms underlying this relationship among healthcare undergraduates.

## 3. Materials and Methods

### 3.1. Study Design, Settings and Participants

A descriptive quantitative research design was adopted by using a self-administered survey to investigate health self-management ability, self-rated health, eHealth literacy and resistance to peer influence of undergraduate healthcare students, and explore their relationships. 

Convenient sampling was adopted to recruit participants from the Faculty of Medicine at a public and comprehensive university located in an east coastal city of Shandong Province. Eligible students included those aged 18 and above, being full-time undergraduates, enrolled in a healthcare program, and who agreed to participate in the study. All undergraduates in this university are full-time and generally reside on campus in a dormitory. Students in the Faculty of Medicine major in seven disciplines, namely, clinical medicine, dentistry, preventive medicine, nursing, pharmacy, medical imaging, and medical laboratory science. The study did not recruit students in the last year of their programs because they engaged in practicing in different clinical settings.

The minimum sample size was calculated using the G*power 3.1.9.2 program for multiple regression analysis. Based on a significance level of 0.05, an intermediate effect size f^2^ of 0.15, power (1 − β) of 0.95, and number of predictors of 13, the resulting sample size was 189. For a multiple mediator model, research suggested that a sample size of 500 was required to detect significant mediating effect if the indirect effect was small [29]. A total of 600 questionnaires were initially distributed to students who were willing to participate in the study between July and September 2021. Finally, 515 valid questionnaires were collected, with an effective response rate of 85.8%. 

The study was approved by Ethics Committee of the University. Consent was provided by participants in the study.

### 3.2. Measures

#### 3.2.1. General Sociodemographic Questionnaire

This questionnaire contained information on age, gender, majors, academic performance, residence, whether they were a single child or not in their family, parental education, times of weekly physical exercise, and body mass index (BMI).

#### 3.2.2. Adults’ Health Self-Management Ability Assessment Scale 

This scale consists of 3 subscales of health self-management behavior (including 14 items in three dimensions of diet self-management, exercise self-management, and disease coping), health self-management cognition (including 14 items in two dimensions of health belief and self-efficacy) and health self-management environment (including 10 items in two dimensions of resource utilization and self-management of environment), with a total of 38 items [30]. A 5-point Likert scale was used to assess health self-management ability which was classified into three levels based on the total score: low level (38–76 points), medium level (77–152 points) and high level (153–190 points). The Cronbach’s α coefficient and content validity of the scale were 0.933 and 0.895, respectively.

#### 3.2.3. The Self Rated Health Measurement Scale (SRHMS)

The SRHMS was developed to assess health for people over 14 years old in China [31]. It consists of 48 items in overall health evaluation dimension and three subscales of self-rated physical health, mental health and social health. The SRHMS adopts a 10-point Likert scale, and a higher score indicates better health status. For comparison, the original raw scores of the total scale and subscales were converted according to the formula: the converted score = (original score − theoretical minimum score)/(theoretical maximum score − theoretical minimum score) × 100. The total Cronbach’s α coefficient of the scale was 0.942.

#### 3.2.4. The eHealth Literacy Scale for College Students

The eHealth Literacy scale for Chinese college students was developed by Tang et al. [32]. The scale includes 20 items in three dimensions, i.e., the eHealth acquisition ability (7 items), the eHealth information evaluation ability (8 items) and the eHealth applying ability (5 items). It uses a 5-point Likert scale to calculate the score, generating a total score range of 20–100. Higher scores indicate higher eHealth literacy. The Cronbach’s α coefficients of the scale and the three dimensions were 0.836–0.915, and the factor load coefficients were 0.451–0.829, demonstrating its good reliability and validity.

#### 3.2.5. The Chinese Version of Resistance to Peer Influence Scale (RPIS)

Resistance to peer influence is conceptualized as an individual’s tendency to resist peer pressure [33]. The Chinese version of RPIS was translated and revised by An [34] to evaluate peer interaction in adolescents and consisted of 10 statements. Each statement represented a peer interaction scenario and was assessed on a 4-point Likert scale. Higher scores indicated greater resistance to peer influence. The Cronbach’s α coefficient of the scale was 0.660. 

### 3.3. Data Analyses

SPSS version 26 software was used for statistical analyses of the data. Descriptive statistics were used to describe the basic information of participants’ characteristics and the scores (e.g., mean and standard deviation) of the study variables. Pearson correlations were calculated between study variables. Multivariate linear regression was conducted to identify the predicting factors of health self-management. Hayes PROCESS macro version 3.5 [35] was used to examine the indirect effects of self-rated health on health self-management through eHealth literacy and resistance to peer influence. Specifically, a serial multiple mediation analysis (model 6) was used to test the indirect effects. The bootstrapping method (5000 resamples) was used to estimate the 95% bias-corrected confidence interval (BC CI) for the indirect effects of each mediator [36]. When the 95% confidence interval does not include zero, it is considered to have significant indirect effects. A *p* value < 0.05 was used to determine statistical significance.

## 4. Results

### 4.1. Common Method Deviation Analysis

Harman’s single factor test was applied to exclude common method deviation caused by the questionnaire method. The results showed that there were 16 factors with eigenvalues greater than 1, and the variation explained by the first factor was 25.11%, which was below the critical value of 40%. This demonstrates that the effect of common method deviation would not influence the data results.

### 4.2. Participant Characteristics

The participants were aged 18–21 years (mean = 19.27, SD = 1.21). The majority of participants were female (76.3%), not a single child in their family (66.8%), and had normal weight (64.1%). Of the participants, 41.3% were majoring in nursing, 37.5% were majoring in clinical medicine, 46.7% were freshmen, 39.0% were sophomores, and 14.3% were juniors. Over half of the participants (62.2%) did physical exercise less than twice weekly. Detailed information is presented in Table 1.

### 4.3. Health Self-Management, Self-Rated Health, Resistance to Peer Influence and eHealth Literacy of Chinese Healthcare Undergraduates

The total score of health self-management of healthcare students was 150.66 ± 23.71, with the mean item scores of health self-management behavior, health self-management cognition and health self-management environment subscales being 3.57 ± 0.74, 4.02 ± 0.71 and 4.44 ± 0.70, respectively. The total score of self-rated health of healthcare students was 77.35 ± 11.94. The scores of self-rated physical health, mental health and social health were 81.00 ± 10.82, 75.77 ± 15.27, and 74.95 ± 15.04, respectively. The total score of resistance to peer influence was 27.56 ± 3.71. The total score of eHealth literacy was 62.97 ± 13.78, with the mean item scores of eHealth acquisition ability, eHealth information evaluation ability and eHealth applying ability dimensions being 3.16 ± 0.79, 3.23 ± 0.75, and 3.01 ± 0.76, respectively.

### 4.4. Bivariate Correlations between Health Self-Management, Self-Rated Health, Resistance to Peer Influence and eHealth Literacy 

Table 2 shows that the four variables were significantly and positively correlated at the 1% level, indicating that further mediation effects could be tested. Specifically, health self-management was positively correlated with self-rated health (*r* = 0.474), with resistance to peer influence (*r* = 0.369) and with eHealth literacy (*r* = 0.357). Self-rated health was positively correlated with both resistance to peer influence (*r* = 0.328) and eHealth literacy (*r* = 0.199). Resistance to peer influence was positively correlated with eHealth literacy (*r* = 0.239). 

### 4.5. Multivariate Regression Analysis on Health Self-Management

Multivariate linear regression analysis was performed to evaluate the predicting factors of health self-management. The results showed that the regression equation was significant (*F* = 57.469, *p* < 0.001) (Table 3). Specifically, self-rated health (*β* = 0.329, *p* < 0.001), eHealth literacy (*β* = 0.220, *p* < 0.001), resistance to peer influence (*β* = 0.171, *p* < 0.001), times of physical exercise per week (*β* = 0.157, *p* < 0.001), and academic performance (*β* = 0.091, *p* = 0.012) were significant predictors of health self-management among healthcare undergraduates, which together explained 35.5% of the total variation.

### 4.6. Mediating Effects of eHealth Literacy and Resistance to Peer Influence

**Figure 1:**
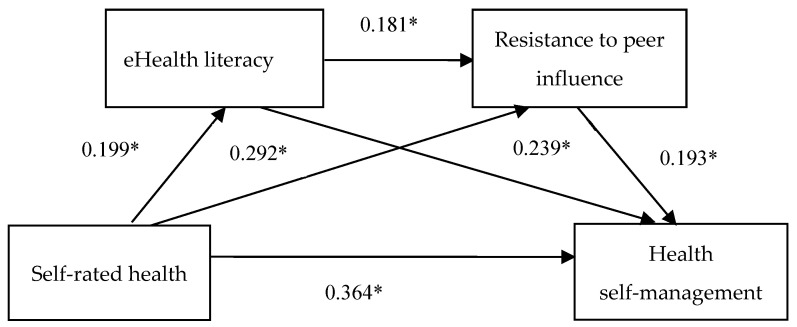
The double chain mediation model of eHealth literacy and resistance to peer influence as mediators of the effect of self-rated health on health self-management. * *p* < 0.001.

The results of bias-corrected percentile bootstrap analysis revealed significant indirect effects of eHealth literacy and resistance to peer influence on the relationship between self-rated health on health self-management (Table 4 and Figure 1). The total effect of self-rated health on health self-management was 0.853 (*SE* = 0.070, *t* = 12.206, *p* < 0.001, 95% CI [0.716, 0.991]). The total direct effect and indirect effect of self-rated health on health self-management was 0.654 (*SE* = 0.069, *t* = 9.402, *p* < 0.001, 95% CI [0.518, 0.791]), and 0.199 (SE = 0.040, 95% CI [0.126, 0.284]), respectively. The indirect effects followed three paths: self-rated health→eHealth literacy→health self-management (estimated effect = 0.085); self-rated health →resistance to peer influence→health self-management (estimated effect = 0.101); and self-rated health→eHealth literacy→resistance to peer influence→health self-management (estimated effect = 0.013). As displayed in Table 4, all the three paths were significant because their 95% CI did not contain zero. The mediation effects of the three paths accounted for 42.71%, 50.75%, and 6.53% of the total indirect effects, respectively. Figure 1 shows the standardized path coefficients of the proposed serial multiple mediation model, representing the direct path coefficients between the variables.

## 5. Discussion

Health self-management is especially important for healthcare undergraduates, as these individuals are at the early adulthood stage and will play an important part in health promotion for the general population. This study identified the associations between self-rated health, eHealth literacy, resistance to peer influence and health self-management, as well as the mediating effects by eHealth literacy and resistance to peer influence. The findings verified the hypothesis that self-rated health had both direct and indirect effects on health self-management. In particular, eHealth literacy and resistance to peer influence played both an independent mediating and serial-multiple mediating roles in the association between self-rated health and health self-management.

In the present study, healthcare undergraduates reported a medium level of health self-management, with the lowest score in the health self-management behavior subscale and highest score in the health self-management environment subscale. This finding indicates that healthcare undergraduates could not adequately translate resources and knowledge into healthy behaviors, which casts light on a gap between cognition and behavior. Pender’s HPM pinpoints that a set of variables of behavioral specific knowledge and affect are of motivational significance for developing health-promoting behaviors [14]. Therefore, affect factors that may play a role in the transformation of knowledge into behavior for healthcare undergraduates remain to be further explored. 

### 5.1. The Direct Effect of Self-Rated Health on Health Self-Management

The present study evinced that self-rated health, eHealth literacy, and resistance to peer influence were positively correlated with health self-management among healthcare undergraduates. Simultaneously, the three variables were the major significant predictors of health self-management in the multivariate regression analysis. This is consistent with Pender’s HPM and some previous research. Self-rated health represents an individual’s subjective perception of their own state of health. Yang et al. [9] found that health concern positively predicted health-promoting lifestyles among college students. Healthcare undergraduates with better self-rated health status may pay more attention to their health and are motivated to manage their health. 

### 5.2. Indirect Effects of Self-Rated Health on Health Self-Management

Notably, the current study uncovered the mechanism of how self-rated health influenced health self-management among healthcare undergraduates. Specifically, eHealth literacy mediated the positive effect of self-rated health on health self-management. The integrative model of eHealth use indicates that intrinsic interest in health is a factor influencing eHealth literacy, and people with eHealth literacy are more inclined to use the internet to find, understand, evaluate and apply health-related information to promote health behaviors [37]. Overall, self-rated health promoted students to obtain and utilize eHealth information, which subsequently raised their intention and empowered them to manage their own health. Previous research found that eHealth literacy directly and indirectly influenced health-promoting behaviors among nursing college students in Korea, in which health information-seeking behavior, social media use and self-care agency played mediating roles [19]. Likewise, healthcare undergraduates possessed higher eHealth literacy in the present study, and had adequate knowledge and ability to make health decisions and to engage in a range of health self-management actions. 

In addition, resistance to peer influence played an independent mediating role, similar to eHealth literacy. For undergraduates at the late adolescence or early adulthood stage, peers serve as a significant source of socialization and exert impact on their attitudes, cognition and health behaviors on account of homophily (i.e., similarities between friends in a dyad or among peers in a network) and for seeking social influence [38]. Previous research demonstrated that resistance to peer influence was a major predictor of health behaviors among healthcare college students [2], and lower resistance to peer influence increased risky decision making [16]. Self-rated health reflects an individuals’ well-being [39], and three aspects of self-rated physical health, mental health and social health were measured in the present study. Better self-rated health signifies students’ overall well-being and their better capability to resist peer pressure. This explains the mediating effect of resistance to peer influence on the relationship between self-rated health and health self-management.

### 5.3. The Chain Mediating Effects

Notably, eHealth literacy and resistance to peer influence played a chain-mediating role in the mechanism of self-rated health affecting health self-management. Namely, self-rated health promoted students’ resistance to peer influence by increasing their eHealth literacy, which, in turn, influenced health self-management. The underlying mechanism could be that students with better ability to search and acquire online information were more capable of utilizing the information to resist peer influence. According to the social ecology theory [12] and health promotion model [9], adolescents and young adults are deeply influenced by peers’ behavior, beliefs, or attitude. To counteract negative peer influence, young people including healthcare undergraduates need some external support. Norman and Skinner defined eHealth literacy as the ability to seek, search, understand, and appraise health-related information from electronic resources and apply the knowledge to address health problems [24]. Therefore, students with higher eHealth literacy could better evaluate health-related online information for making informed decision in a networked world. The chain mediating results of the study demonstrated that eHealth literacy played a protective role in resisting peer pressure. This further suggests that efforts are needed to promote eHealth literacy of healthcare undergraduates who actively engage in digital technologies so they can be competent to resist peer pressure and simultaneously improve their health self-management.

### 5.4. Practical Implications

The study results provide important implications for educators and policymakers in medical institutions. Firstly, the health self-management ability of healthcare undergraduates needs to be enhanced, particularly in the domain of health self-management behavior. In an era of network information, the use of modern technology has shown promise in promoting healthcare professionals’ skills of supporting patients’ self-management [40]. Internet-based learning can disseminate knowledge via multimedia with much convenience and privacy. Therefore, eLearning may be an alternative approach to improving health self-management for healthcare undergraduates. A meta-analysis revealed that eLearning was at least as effective as traditional learning approaches. The benefits of eLearning included increased accessibility to education, improved self-efficacy, knowledge generation, cost effectiveness, learner flexibility and interactivity that improved healthcare professional behavior or skill development [41]. However, a recent study reported that eLearning delivery mode showed less efficacy than videoconferencing and in-person hybrid in the World Health Organization’s caregiver training program, in terms of increasing well-being of caregivers of children with autism spectrum disorder and mitigating children’s challenging behaviors [42]. The eLearning caregivers in the study reported lack of feedback and interactive activities, and experiencing loneliness that partially generated their frustration. Simultaneously, eLearning mode seemed more suitable for caregivers who were not quite as busy and had better time management skills [42]. Another meta-analysis study also suggested that blended learning, a combination of eLearning and face-to-face methods, was valued to support patient self-management skills development for healthcare professionals due to the importance of subsequent practice and reflection following learning activities [40]. These studies indicate that acceptability, feasibility, effectiveness and modes are critical considerations when designing and implementing eLearning programs for healthcare undergraduates.

In view of the mediating effects of eHealth literacy and resistance to peer influence, interventions such as educational programs designed to increase eHealth literacy and resistance to peer influence are essential to boost health self-management among healthcare undergraduates. A study revealed a positive correlation between eHealth literacy and leaning experiences of the six domains (traditional, media, health, computer, scientific, and information literacy) of eHealth literacy among Japanese undergraduate nursing students [43]. Therefore, healthcare students need to be provided with opportunities to systematically learn about searching, assessing, selecting, and applying high-quality eHealth information in the curriculum. 

In the meantime, strengthening healthcare undergraduates’ levels of confidence in resisting peer influence is a worthwhile intervention strategy, which can be designed and tailored by incorporating aspects of social skills training to resist negative peer pressure [44]. 

### 5.5. Limitations

Some potential limitations of the study should be considered during interpretation of the findings. First, the sample did not include senior undergraduates and the findings, thus, may not be generalizable to the diverse healthcare undergraduates in China. Second, all variables in the study were measured using self-reporting scales, which may lead to some potential social desirability response bias when estimating the associations. Additionally, this study only tested two mediators while other psychosocial variables such as self-efficacy and social support may exert mediating effects, as demonstrated by previous research on different populations [45,46,47]. Moreover, some potential covariates such as lifestyle and medical history may indirectly affect healthcare undergraduates’ eHealth literacy, health management, and behavior, as reported by previous studies [9,48], while the present study did not include these variables. Future study could consider adding these variables to examine their effects. Lastly, the nature of cross-sectional design limits the interpretation of causality among self-rated health, eHealth literacy, resistance to peer influence and health self-management. Despite the limitations, to our knowledge, this study is one of the first examining associations between self-rated health, eHealth literacy, resistance to peer influence and health self-management of healthcare undergraduates. The present study provides insights into the underlying mechanisms of the link between self-rated health and health self-management and highlights the distinct mediating roles of eHealth literacy and resistance to peer influence.

## 6. Conclusions

Self-rated health directly and indirectly affects healthcare undergraduates’ health self-management through the mediating effect of eHealth literacy and resistance to peer influence. The findings contribute to a better understanding of the mechanism of how self-rated health influences the health self-management of healthcare undergraduates. Interventions targeting eHealth literacy and resistance to peer influence could be implemented to promote students’ health self-management and further improve their ability of supporting clients for optimal health self-management in their future practice.

## Figures and Tables

**Table 1:** Participants’ demographic characteristics (N = 515).

Variables	Categories	*n* (%)
Major	Nursing	213 (41.3)
	Clinical medicine	193 (37.5)
	other ^a^	109 (21.2)
Year of study	Freshman	240 (46.6)
	Sophomore	201 (39.0)
	Junior	74 (14.4)
Gender	Male	122 (23.7)
	Female	393 (76.3)
Residence	Rural	298 (57.9)
	Urban	217 (42.1)
Single child in the family	Yes	171 (33.2)
	No	344 (66.8)
Father’s education level	Middle school and below	262 (50.9)
	High school and equivalent	127 (24.7)
	College and above	126 (24.5)
Mother’s education level	Middle school and below	295 (57.3)
	High school and equivalent	105 (20.4)
	College and above	115 (22.3)
Academic performance	Below average	121 (23.5)
	At average	197 (38.3)
	Above average	197 (38.2)
Weekly physical exercise	<2 times	320 (62.2)
	3–4 times	144 (28.0)
	>5 times	51 (9.9)
BMI ^b^	Underweight	126 (24.5)
	Normal	330 (64.1)
	Overweight	59 (11.4)

Note. ^a^ Other majors including stomatology, medical laboratory technology, medical imaging, public health, pharmacy. ^b^ BMI = body mass index; BMI was classified into the three categories, underweight (<18.5), normal (18.5–24.9), and overweight (≥25 kg/m^2^).

**Table 2:** Correlations between health self-management, resistance to peer influence, eHealth literacy and self-rated health.

Variables	Health Self-Management	Self-Rated Health	Resistance to Peer Influence	eHealth Literacy
Health self-management	1			
Self-rated health	0.474 *	1		
Resistance to peer influence	0.369 *	0.328 *	1	
eHealth Literacy	0.357 *	0.199 *	0.239 *	1

* *p* < 0.001.

**Table 3:** Multivariate linear regression analysis of health self-management ability among Chinese healthcare undergraduates.

Variables	Unstandardized Coefficient (B)	Standard Error (SE)	Standardized Coefficient (β)	*t*	*p*
Constant	45.896	8.504		5.397	<0.001
Self-rated health	0.591	0.069	0.329	8.533	<0.001
eHealth literacy	0.379	0.064	0.220	5.926	<0.001
Resistance to peer influence	1.121	0.252	0.171	4.446	<0.001
Weekly physical exercise	5.549	1.304	0.157	4.254	<0.001
Academic performance	2.779	1.104	0.091	2.516	0.012

*F* = 57.469, *p* < 0.001, *R* = 0.601, *R*^2^ = 0.361, adjusted *R*^2^ = 0.355.

**Table 4:** Total, direct and indirect effects in the multiple mediator model.

Model	Estimated Effect	Boot SE	95% BC CI	Total Mediation Effect
Total effect of X on Y	0.853	0.070	0.716–0.991	-
Total direct effect of X on Y	0.654	0.069	0.518–0.791	-
Total indirect effect of X on Y	0.199	0.040	0.126–0.284	23.30%
X→M1→Y	0.085	0.030	0.034–0.153	9.96%
X→M2→Y	0.101	0.027	0.055–0.157	11.84%
X→M1→M2→Y	0.013	0.005	0.004–0.024	1.52%

X, self-rated health; M1, eHealth literacy; M2, resistance to peer influence; Y, health self-management.

## Data Availability

The data that support the findings of this study are available from the corresponding author upon request.
